# Foot health of Aboriginal and Torres Strait Islander Peoples in regional and rural NSW, Australia

**DOI:** 10.1186/s13047-020-00397-w

**Published:** 2020-05-28

**Authors:** Matthew West, Sean Sadler, Fiona Hawke, Shannon E. Munteanu, Vivienne Chuter

**Affiliations:** 1 Discipline of Podiatry, University of Newcastle, Ourimbah, NSW 2258 Australia; 2 Discipline of Podiatry, School of Allied Health, Human Services and Sport, La Trobe University, Melbourne, Victoria 3086 Australia; 3 La Trobe Sport and Exercise Medicine Research Centre, School of Allied Health, Human Services and Sport, La Trobe University, Melbourne, Victoria 3086 Australia; 4 Priority Research Centre for Physical Activity and Nutrition, University of Newcastle, Newcastle, NSW 2308 Australia

**Keywords:** Indigenous peoples, Foot health status questionnaire, Podiatry, Cultural competency, Prevention

## Abstract

**Background:**

Foot health of Aboriginal and Torres Strait Islander Australians’ has not been established. Additionally, studies have shown that there is a lack of engagement of this population with general preventive foot care services. The aim of this study was to establish foot health in Aboriginal and Torres Strait Islander people attending two recently developed, culturally safe podiatry services in rural and regional New South Wales (NSW), Australia. Secondarily the relationship between self-perceived foot health and some medical and demographic characteristics was investigated.

**Methods:**

This descriptive cross-sectional study included participants attending the culturally safe foot health care services managed by the University of Newcastle on the Central Coast or in Wellington, both located in NSW, Australia. At the consultation, participants completed the Foot Health Status Questionnaire (FHSQ) with the assistance of an Aboriginal health care worker, underwent basic vascular and neurological screening, and podiatric treatment.

**Results:**

A total of 111 Aboriginal and Torres Strait Islander Australians (48 from the Central Coast, and 63 from Wellington) were included. FHSQ scores for pain (75.7 ± 26.8), function (80.2 ± 25.2), footwear (53.9 ± 33.4), and general foot health (62.0 ± 30.9) were generally good, but below the optimal score of 100. The presence of diabetes (*n* = 39 of 111 participants or 35.1%) was associated with lower levels of self-perceived foot function (r = − 0.20, *n* = 107, *p* = 0.04).

**Conclusion:**

We found that community-based foot health care services that are culturally safe are utilised by Aboriginal and Torres Strait Islander Peoples not currently at high risk of foot complications. This supports the use of culturally safe foot care services to improve engagement with preventative foot care. Future research should continue to be driven by Aboriginal and Torres Strait Islander Peoples and investigate ways to implement additional screening measures and undertake prospective evaluation of the impact of such services on health related outcomes in these communities.

## Background

Aboriginal and Torres Strait Islander Australians have a five-to-six-fold increased likelihood of developing foot complications including foot ulcer and amputation compared to non-Indigenous Australians [1–4]. Similarly, this population experiences a four-fold increase in risk of peripheral neuropathy, and are more likely to have peripheral arterial disease but less likely to have had it diagnosed [1–4]. This problem is exacerbated in rural and regional Australia where there is limited health care service availability and poor engagement with existing services [3, 5]. Clustering of health risk factors, including disproportionately high rates of diabetes, greater risk of vascular disease, reduced socioeconomic circumstances, reduced health literacy, lack of access to culturally safe care, poor engagement with preventative health care services, and lifestyle factors including high rates of smoking and poor nutrition have been proposed to increase the risk of foot complications in this population even further [6].

Despite the documented high rates of foot complications in Aboriginal and Torres Strait Islander Peoples in tertiary healthcare settings throughout Australia, there are remarkably little data describing foot health at a community level. The available evidence indicates there is poor engagement with early intervention foot care services [3, 5]. Individuals typically seek help once a foot problem is present and often of a complex nature e.g. foot ulceration or infection, and this contributes to high rates of hospitalisation and amputation [5, 7, 8]. The lack of descriptive data relating to foot health status in this population at a community level limits our understanding of service provision needs. Additionally, the paucity of available data also limits our understanding of Aboriginal and Torres Strait Islanders’ perceptions of health and illness, which may be a key driver in their health seeking behaviours. For example, previous research in an Aboriginal population in remote Arnhem Land found that community members’ concepts of health related to whether they could carry out daily activities and live with their family, regardless of diagnosis of disease [9]. Therefore, the primary aim of this study was to determine the point prevalence of foot health of Aboriginal and Torres Strait Islander Australians presenting to culturally safe podiatry services in a rural and a regional community of New South Wales (NSW), Australia. Secondarily, we aimed to explore the association between demographic variables (age and sex), smoking status (never or past/current), diabetes status (present or absent) and foot health.

## Methods

The University of Newcastle Human Research Ethics Committee (H-2018-0035) granted ethics approval and the Australian Health and Medical Research Council approved the project (1376/18). Written informed consent was obtained from all participants at the start of the consultation.

### Participants and settings

This descriptive cross-sectional study recruited Aboriginal and Torres Strait Islander People aged 18 years and older presenting to one of two culturally safe foot health services between March 2018 and July 2019. One of the clinics is located at Wyong hospital, which is on the Central Coast of NSW, approximately 100 km north of Sydney, Australia. The other clinic is located in Wellington, which is a town in inland NSW and is approximately 360 km North West of Sydney, Australia. Both clinics are managed by the University of Newcastle, Discipline of Podiatry and are designed to be culturally safe community clinics that are led by an Aboriginal Podiatrist, supported by Aboriginal Health Worker, and provide student placements. In being culturally safe, these clinics are designed to create an environment that is considerate of the spiritual, physical, social, and emotional world view of Aboriginal and Torres Strait Islander people, thereby creating a clinical experience which is conducive to, and supportive of, the specific needs of this community [10]. Additionally, the approach to management of patients within these clinics is one that recognises the importance of culture, family, and community for Aboriginal and Torres Strait Islander people. The services provide a mix of podiatric clinical care and health promotion with a focus on diabetes related foot complications and prevention education. The Central Coast service operates on a weekly basis from the University of Newcastle Podiatry Clinic, and the Wellington Service via an Outreach program which occurs 3 to 4 times per year in a local Aboriginal community facility. The latter service operates as a drop-in clinic without any formal appointment times.

### Participant characteristics

Participant characteristics were self-reported by participants on a generic questionnaire developed by the authors for this study and included age, sex, smoking status (never or past/current), and diabetes mellitus status (present or absent).

### Measurement of foot health

Following measurement of participants’ characteristics, their foot health was measured through the use of a (i) patient reported outcome measure, the Foot Health Status Questionnaire (FHSQ), and (ii) clinical assessment.

#### Foot health status questionnaire

Foot-specific health-related quality of life was evaluated using the FHSQ. The FHSQ is a 13-item questionnaire used to evaluate foot health across four domains (pain, function, footwear and general foot health) and can be accessed in full online via the FHSQ website [11]. Questions in each domain are scored using a 5-point Likert scale. Responses were then transformed into domain scores, that ranged from 0 (worst foot health) to 100 (best foot health). The FHSQ [11] is a valid questionnaire that has shown high retest reliability [12]. The FHSQ was not specifically designed for Aboriginal and Torres Strait Islander Peoples and may not consider how cultural differences might influence responses. To limit the potential impact of this an Aboriginal health care worker was present to assist participants if they had difficulty understanding the questions.

#### Clinical foot assessment

Clinical foot assessment was then conducted and involved neurological and vascular assessment of both feet and was performed by registered podiatrists with relevant clinical experience. Neurological assessment was performed via the 10-site monofilament test using a 5.07 Semmes-Weinstein monofilament [13]. An abnormal result was defined as a participant identifying less than seven out of ten sites on any foot [14]. Vascular assessment was performed via a Doppler assessment of the dorsalis pedis and posterior tibial arteries [15], using a bi-directional hand-held Doppler (Hadeco© ES-100 V3 8 mHz). An abnormal result was defined as the presence of monophasic waveforms for either artery of the left or right foot [16]. Studies have demonstrated that both monofilament [13] and Doppler waveform assessment [16] have acceptable levels of reliability.

### Statistical analyses

Data were entered into Microsoft Excel and the FHSQ software (version 1.03) and then exported to the statistical package for the social sciences (SPSS) program (version 25.0 Chicago, Illinois, USA) for analysis. For continuous data, data were assessed for normality prior to inferential analysis. For descriptive analysis, means and standard deviations are reported for continuous variables, and frequency and percentages for dichotomous variables.

Correlations were performed to determine the level of association between demographic factors (age and sex), smoking status (never or past/current), and diabetes status (present or absent) for each of the FHSQ domains. The strength of the correlation was interpreted as small (r = 0.10 to 0.29), moderate (r = 0.30 to 0.49), and large (r = 0.50 to 1.0) [17]. A standard linear regression was performed to determine the proportion of variance in each domain of the FHSQ attributed to a significantly (*p* < 0.05) correlated independent variable. Assumptions for the analyses were met.

## Results

### Participants

A total of 111 Aboriginal Australians, 48 from the Central Coast and 63 from Wellington enrolled in this study (Table 1). These figures represent approximately 0.4 and 5.6% of the total number of Aboriginal and Torres Strait Islander people living in the Central Coast and Wellington areas.

**Table 1:** Participant characteristics

	Total group	Central Coast	Wellington
Age, mean (SD)	52.5 (16.3)	53.9 (15.3)	51.3 (17.0)
Not reported by participant, n (% of total)	10 (9.0%)	1 (0.9%)	9 (8.1%)
Sex			
Female, n (% of total)	57 (51.4%)	20 (41.7%)	37 (58.7%)
Male, n (% of total)	53 (47.7%)	28 (58.3%)	25 (39.7%)
Not reported by participant, n (% of total)	1 (0.9%)	0 (0.0%)	1 (1.6%)
Smoking status			
Never, n (% of total)	41 (36.9%)	20 (41.7%)	21 (33.3%)
Past or current, n (% of total)	57 (51.4%)	28 (58.3%)	29 (46.0%)
Not reported by participant, n (% of total)	13 (11.7%)	0 (0.0%)	13 (20.7%)
Diabetes status			
No diabetes, n (% of total)	68 (61.3%)	28 (58.3%)	40 (63.5%)
Diabetes, n (% of total)	39 (35.1%)	20 (41.7%)	19 (30.1%)
Not reported by participant, n (% of total)	4 (3.6%)	0 (0.0%)	4 (6.4%)

### Foot health assessment

Findings from the foot health assessments are shown in Table 2. The vascular and neurological status of a number of participants is unknown because some participants chose not to have the assessments performed or only had part of the assessments conducted (Table 2).

**Table 2:** Foot health assessment

Foot health measure	Total group mean ± SD (range), *n* = 111	Central Coast mean ± SD (range) *n* = 48	Wellington mean ± SD (range) *n* = 63
FHSQ, pain	75.7 ± 26.8 (0–100)	77.5 ± 27.3 (0–100)	74.4 ± 26.5 (0–100)
FHSQ, function	80.2 ± 25.2 (0–100)	83.1 ± 21.1 (25–100)	78.0 ± 27.9 (0–100)
FHSQ, footwear	53.9 ± 33.4 (0–100)	58.3 ± 31.7 (0–100)	50.7 ± 34.6 (0–100)
FHSQ, general foot health	62.0 ± 30.9 (0–100)	66.9 ± 30.6 (0–100)	58.3 ± 30.8 (0–100)
Monofilament			
Normal^a^, n (% of total)	63 (56.8%)	42 (87.5%)	21 (33.3%)
Abnormal^b^, n (% of total)	4 (3.6%)	2 (4.2%)	2 (3.2%)
Missing, n (% of total)	44 (39.6%)	4 (8.3%)	40 (63.5%)
Doppler			
Normal^c^, n (% of total)	60 (54.1%)	38 (79.2%)	22 (34.9%)
Abnormal^d,^ n (% of total)	4 (3.6%)	2 (4.2%)	2 (3.2%)
Missing, n (% of total)	47 (42.3%)	8 (16.6%)	39 (61.9%)

^a^Defined has ≥7/10 both feet. ^b^Defined has < 7 on one foot or more. ^c^Defined has no monophasic waveform. ^d^Defined has one or more monophasic waveform/s

All FHSQ domain scores were notably less than the optimum score of 100 (Table 2), with the footwear and general foot health domains being lowest. There were no statistically significant differences in FHSQ domain scores between regions. Additionally, there were no differences in rates of overt peripheral arterial disease or peripheral neuropathy between the regions (Table 2).

### Associations between participant characteristics and FHSQ domains

Being female was significantly associated with lower scores on the footwear domain (r = 0.29, *n* = 110, *p* < 0.01). The presence of diabetes was associated with lower levels of self-perceived foot function (r = − 0.20, *n* = 107, *p* = 0.04) (Table 3).

**Table 3:** Pearson correlation between each FHSQ domain and participant characteristics

	Foot Pain	Foot Function	Footwear	General foot health
Age, *n* = 101	−0.04	−0.17	− 0.09	0.10
Sex, n = 110	0.17	0.15	0.29^**^	−0.04
Diabetes, n = 107	−0.18	− 0.20^*^	− 0.15	−0.01
Location, n = 111	−0.06	−0.10	− 0.11	−0.14
Smoking, *n* = 98	−0.003	−0.05	0.17	−0.10

^*^p < 0.05, ^**^p < 0.01

#### Regression analysis

For the footwear domain, female sex was able to predict 9.0% of the variance in the score for this domain (r^2^ 0.09, *p* < 0.01, beta coefficient 0.29). Linear regression demonstrated that the presence of diabetes predicted 4.0% of the variance in the function domain score (r^2^ 0.04, *p* = 0.04, coefficients beta − 0.20). No significant associations were found between the variables measured and the foot pain and general foot health domains of the FHSQ.

## Discussion

The primary aim of this study was to describe the point prevalence of foot health of Aboriginal Australians presenting across two culturally safe foot health services in rural and regional NSW, Australia. We found that a high proportion of patients reported having diabetes (Central Coast 41.7%, Wellington 30.1%) and a current or past history of smoking (Central Coast 58.3%, Wellington 46.0%). Foot-specific health-related quality of life, evaluated using the FHSQ, was less than the optimum score of 100, with the footwear and general foot health domains being notably poorer than pain and function domains. The neurological and vascular assessments demonstrated that the rates of clinically overt peripheral arterial disease (Central Coast 4.2%, Wellington 3.2%) and peripheral neuropathy (Central Coast 4.2%, Wellington 3.2%) were low. There were not any regional differences for foot-specific health-related quality of life or overt peripheral arterial disease or peripheral neuropathy.

Capacity for comparison of the FHSQ data from this current study with previous research is challenging as there are no published data relating to foot-specific health-related quality of life in Aboriginal and Torres Strait Islander Australians. Much of the existing data investigating FHSQ domains in the general population relate to specific pathologies and the outcome of interventions, for example, treatments for plantar heel pain or foot osteoarthritis [18–21]. In the present study, mean scores for the pain, function, footwear, and general foot health domains were all higher than those previously reported in a population of older Australians using podiatry care [22]. The comparatively high FHSQ scores for our study population suggests that, on average, participants felt healthy and perceived their feet as healthy and this was consistent with our low rates of abnormal vascular and neurological findings.

Existing data relating to foot heath in Aboriginal Australians indicates high rates of disease, including peripheral vascular disease and neuropathy, with many of the studies derived from outcomes from public hospital admissions and high risk foot centres [3, 4, 23]. Importantly the findings of this present study should not be considered contradictory to previous research. The high rates and devastating outcomes of foot disease for Aboriginal and Torres Strait Islander Australians are unequivocal [2, 4, 24, 25]. Our research outcomes most likely reflect recruitment from a community-based population and offer insight into the potential effectiveness of providing community-led, culturally safe health care to reduce morbidity and mortality through early intervention, and of the critical role of accessible prevention and management services.

Lack of engagement of Aboriginal and Torres Strait Islander Peoples with existing preventative foot care services, for example those with diabetes, is common [5, 8] and needs to be improved to successfully reduce foot disease and complications such as amputations [26]. A number of barriers to Indigenous populations accessing preventative care have been identified, including poor relationships with health care providers, health care providers’ lack of acceptance of the role of family in care provision, and poor community engagement with available services [9, 27–29]. In addition, fatalist beliefs towards one’s own health (i.e. that ill-health is unavoidable) and towards Western health service provision (i.e. that accessing health care is only for the very sick and has negative outcomes) has been documented in Australian Aboriginal and Torres Strait Islander Peoples [9]. This belief system has been linked to historical and current issues of dispossession and socioeconomic inequality, concern over being removed from family and community for treatment, along with lack of improvement in Aboriginal and Torres Strait Islander health outcomes through a Western model of health care delivery. The results of the current study, showing that foot-specific health-related quality of life in the sample was, on average, generally good, with low rates of peripheral arterial disease and neuropathy, provide evidence to refute a fatalistic approach to foot ill-health.

Access to culturally safe health services using Aboriginal health workers provided in spaces that have significance (e.g. cultural or physical) to the local community has been shown to increase utilisation of health care [30–32]. Although a number of initiatives have been developed to provide culturally safe foot care, there has been little evaluation of their effectiveness [7]. Our findings support the use of culturally safe service provision within the community to encourage engagement with and early uptake of preventative care in those with chronic disease. However, further research is needed to broaden the understanding of the fundamental foot health care needs of Aboriginal and Torres Strait Islander Peoples, and to establish effective strategies for preventing foot related complications of chronic disease. Importantly, this must be driven by Aboriginal and Torres Strait Islander Peoples so that the services are not only culturally appropriate and safe, but to help encourage and empower other community members to seek out and engage with health care services in the future.

### Limitations

A number of limitations should be considered when interpreting our findings. We only measured a small number of variables related to participant demographics, diagnosed conditions (only diabetes), and vascular and neurological function. Additional data related to participants’ physical characteristics (e.g. height, weight, and waist circumference), diagnosed conditions (e.g. duration, complications, and disease specific levels of severity such as HbA1c), and additional vascular (e.g. toe and ankle brachial indices) and neurological (e.g. graduated tuning fork) assessments would provide further insight into Aboriginal and Torres Strait Islander Peoples’ lower limb health. Furthermore, the FHSQ has not been validated in Aboriginal and Torres Strait Islander communities and, although administered with the help of an Aboriginal Health Worker, is a Western mechanism to quantify foot-specific health-related quality of life. Further investigation of Aboriginal and Torres Strait Islander perceptions of foot health and what good health entails is required to continue to develop effective foot complication prevention programs. Finally, the vascular or neurological status of the number of participants (40%) who chose not to have assessments performed or for whom assessments were incomplete or were not the participant’s priority is unknown. Although a limitation of the research, this approach was integral to the overarching priority of the clinical services to, first and foremost, develop community trust and engagement. Caution is recommended when attempting to generalise the findings from this study to other Aboriginal and Torres Strait Islander communities, especially those in remote locations, because of potential differences in a number of variables measured and the culturally diverse nature, kinship, and beliefs of these people across all First Nations.

## Conclusions

We found that Aboriginal Australians presenting to recently developed and culturally appropriate podiatry services have relatively high levels of foot health. This suggests that these services provide a unique early opportunity for podiatrists to identify, manage, and implement preventative health care, to minimise the burden of foot complications in this population. Future research should further explore foot health from the perspective of Aboriginal and Torres Strait Islander Peoples and prospectively evaluate the impact of culturally safe foot health services on health outcomes in these communities.

## Data Availability

Requests for further detail on the data collected in this study, or data sharing arrangements, can be submitted to Vivienne Chuter (Vivienne.chuter@newcastle.edu.au).

## Abbreviations

NSW: New South Wales
FHSQ: Foot Health Status Questionnaire
SPSS: Statistical package for social sciences
SD: Standard deviation
